# Narcolepsy: immunity, neural circuitry, and brain network reconfiguration

**DOI:** 10.3389/fimmu.2026.1864667

**Published:** 2026-06-23

**Authors:** Jiajia Chen, Ying Wang, Ying Bai, Youde Cai

**Affiliations:** 1 Department of Sleep Medicine, Guizhou Provincial Second People’s Hospital, Guiyang, China; 2 Department of Neurology, Jinyang Hospital of Guizhou Medical University, Guiyang, China; 3 Graduate School, Guizhou University of Traditional Chinese Medicine, Guiyang, China

**Keywords:** autoimmunity, brain network, hypocretin, narcolepsy, neural circuit

## Abstract

**Introduction:**

Narcolepsy is a rare sleep disorder characterized by cataplexy and excessive daytime sleepiness. The hallmark neuropathological feature is the selective loss of hypothalamic hypocretin (HCRT) neurons. This review systematically summarizes the roles of neuroimmune dysregulation, HCRT neuron loss, and neural circuit remodeling in the pathogenesis of narcolepsy.

**Methods:**

We searched PubMed, Web of Science, and Scopus from 1990 through April 2026. Search terms included narcolepsy, hypocretin/orexin, autoimmunity, HLA, T cell, neural circuit, brain network, and functional connectivity. Articles were included if they were in English, peer-reviewed, and provided mechanistic insights. In addition, key references identified in the search articles were included.We organized the review according to a conceptual framework linking immune dysregulation, hypocretin neuron loss, sleep–wake circuit imbalance, and large-scale brain network reconfiguration.

**Results:**

Genetic susceptibility and environmental triggers collectively promote an immune-mediated attack, causing irreversible loss of lateral hypothalamic HCRT neurons. This disruption impairs sleep-wake circuit homeostasis and upsets the balance between REM-off and REM-on neurons. Consequently, brain network remodeling and functional instability ensue, manifesting as excessive daytime sleepiness, cataplexy, and cognitive impairment.

**Discussion:**

This review provides a theoretical framework integrating immunity, neural circuitry, and brain network reconfiguration in narcolepsy pathophysiology. Understanding these mechanisms may identify potential diagnostic biomarkers and therapeutic targets for narcolepsy, particularly for type 1 narcolepsy.

## 1 Introduction

Narcolepsy is a lifelong, disabling sleep disorder characterized by excessive daytime sleepiness, cataplexy, sleep disruption, hallucinations, and sleep paralysis (1). Orexins (also called hypocretins) are excitatory hypothalamic neuropeptides with two subtypes—orexin A (hypocretin−1) and orexin B (hypocretin−2)—that regulate sleep and wakefulness. According to the third edition of the International Classification of Sleep Disorders (ICSD−3), narcolepsy is divided into type 1 (NT1) and type 2 (NT2). NT1 is characterized by cataplexy and HCRT deficiency, whereas NT2 typically occurs without cataplexy and generally lacks evidence of HCRT deficiency (2). The prevalence of narcolepsy ranges from 0.02% to 0.05%, showing substantial heterogeneity across geographic regions and ethnic groups. Japan has the highest reported prevalence worldwide, with 160–180 cases per 100,000 people. Whether this pronounced ethnic disparity is attributable to the distribution of the human leukocyte antigen (HLA)−DQB1*06:02 allele remains inconclusive. Notably, the carrier frequency of HLA−DQB1*06:02 in the general Japanese population is similar to that in other ethnic groups. This finding suggests that the high prevalence in Japan may involve additional genetic backgrounds, environmental triggers, or immunomodulatory factors. Large−scale population studies are warranted to elucidate these factors (3, 4).

Although narcolepsy is classified as a rare disease, its pathological process encompasses peripheral immune activation, selective neuronal loss, and large−scale functional reorganization of brain networks. In genetically susceptible individuals, exposure to specific environmental triggers leads to T−cell−mediated autoimmune destruction of HCRT neurons. Loss of these neurons triggers compensatory functional reorganization across brain networks, ultimately manifesting as core clinical symptoms, including EDS and cataplexy. Several key questions regarding the disease mechanisms remain unresolved. This review systematically synthesizes the pathogenesis of narcolepsy from three perspectives: immunity, neural circuits, and brain network remodeling.

## 2 Immune injury: the initiating step in pathogenesis

**Figure 1:**
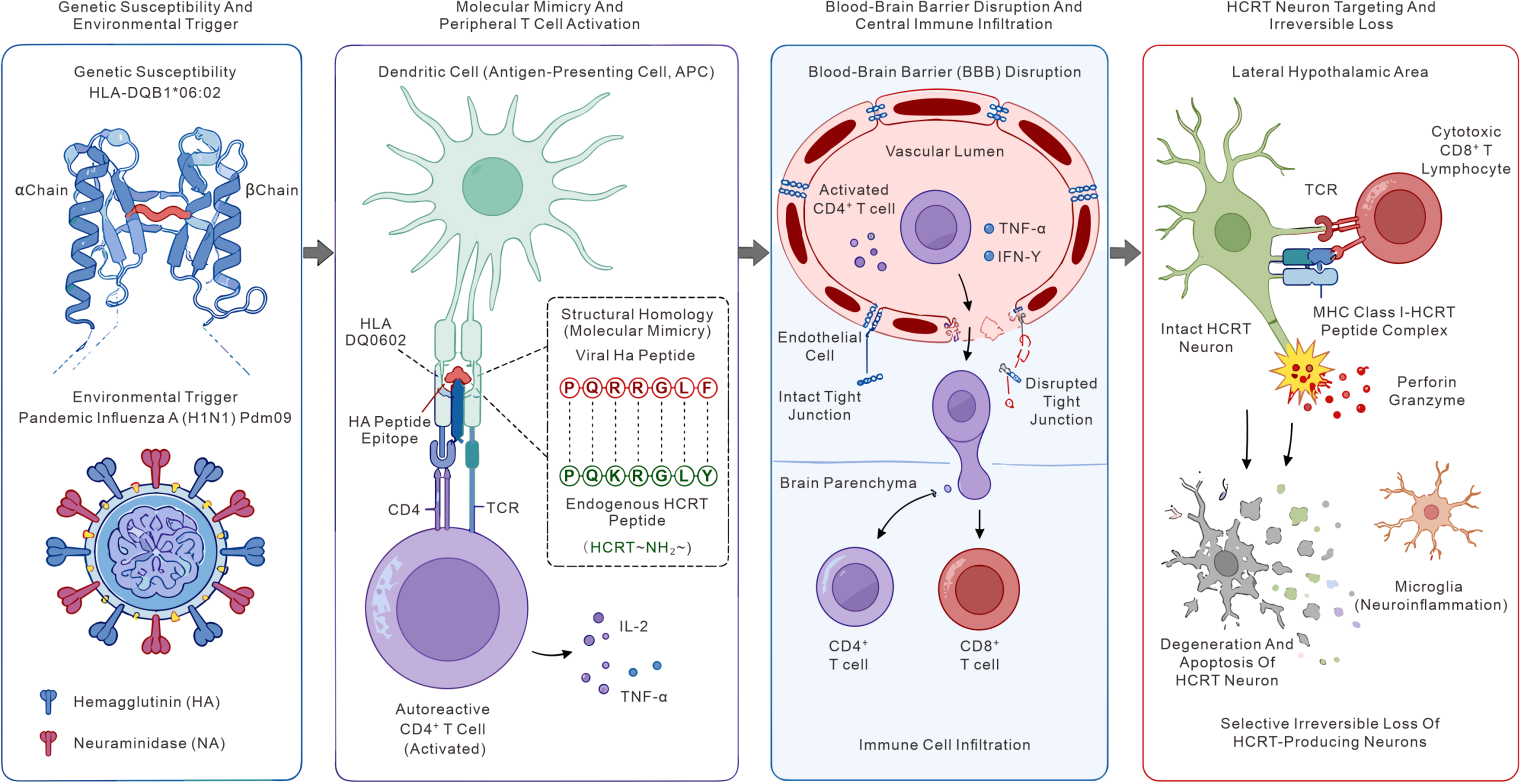
Autoimmune cascade mediating selective hypocretin (HCRT) neuronal loss in type 1 narcolepsy (NT1). Stage 1: Individuals carrying high-risk HLA-DQB1*06:02 are exposed to H1N1 pdm09 influenza virus. Stage 2: Viral hemagglutinin (HA) peptide is presented by antigen-presenting cells (APCs). Molecular mimicry activates autoreactive CD4^+^ T cells to secrete pro-inflammatory cytokines. Stage 3: Cytokines damage the blood-brain barrier (BBB), allowing CD4^+^ and CD8^+^ T cells to infiltrate the hypothalamus. Stage 4: CD8^+^ T cells kill hypocretin (HCRT) neurons via perforin/granzyme, causing irreversible neuronal loss.

Immune injury signifies the fundamental initiating event in the pathogenesis of narcolepsy, which is essentially an autoimmune pathological process triggered by environmental factors against a background of genetic susceptibility, with synergistic involvement of multiple immune pathways. This process is rooted in HLA-related genetic predisposition. In response to external environmental immune stimuli, the body initiates a sequence of immune attacks and amplifies inflammatory effects through the humoral immune system. This process culminates in the selective and irreversible loss of HCRT neurons, which directly induces the core clinical symptoms of narcolepsy (**Figure 1**).

### 2.1 Genetic susceptibility and HLA association

The strong association between narcolepsy and the human leukocyte antigen (HLA) region provides key genetic evidence supporting its immunopathological mechanism. In North American Caucasian populations, the HLA−DQB1*06:02 allele is present in up to 95% of patients with narcolepsy with cataplexy, whereas its frequency is below 40% in those without cataplexy. This cross−ethnic, specific genetic marker points directly to selective, irreversible loss of HCRT neurons, genetically implicating autoimmune attack as the core pathological basis of NT1 (5, 6). This allele typically forms the DQ0602 heterodimer with HLA−DQA1*01:02 and, together with HLA−DRB1*15:01, constitutes the DRB1*15:01−DQA1*01:02−DQB1*06:02 haplotype, the strongest genetic risk unit for NT1 (7). Beyond the allelic level, specific amino acid polymorphisms in the HLA−DQB1 protein receptor epitope also contribute to disease susceptibility. Specifically, residues Ser182 and Thr185 on the DQB1 β−chain are associated with increased risk, whereas Asn182 confers a protective effect (8, 9). In addition, HLA−DQB1*03:01 increases the risk of NT1, whereas protective alleles include DQB1*06:01, DQB1*05:01, DQB1*06:03, and DQA1*01. Given the low frequency of this allele in patients without cataplexy, its testing is no longer used for clinical diagnosis (10). Nevertheless, the allele retains value in predicting individual sleep patterns, especially under conditions of sleep deprivation. Furthermore, clinical improvement observed after immunoglobulin and prednisone intervention further supports the immunopathological hypothesis (11, 12).

Most NT1 cases are sporadic; however, first−degree relatives have a 10− to 40−fold higher risk than the general population, and familial cases more often lack HLA−DQB1*06:02, suggesting involvement of additional genetic factors. Genetic risk profiles differ between early−onset and late−onset patients; polymorphisms in CCR1/CCR3 and HLA−DQB1 are more common in early−onset cases (13). Genome−wide association studies have identified multiple non−HLA immune−related genes, including the T−cell receptor alpha locus, the purinergic receptor P2RY11, cathepsin H, and tumor necrosis factor superfamily member 4. The CPT1B/CHKB gene has been implicated in REM sleep regulation, although its association exhibits racial heterogeneity (14).Mutations in the DNMT1 gene are associated with a rare familial form of narcolepsy accompanied by deafness and cerebellar ataxia (15, 16). Other candidate loci await validation.

### 2.2 Environmental triggers

Epidemiological evidence indicates a strong association between environmental exposures and the risk of NT1. Like sleep disorder phenotype resembling NT1 (17). The marked increase in NT1 incidence following the 2009 H1N1 influenza pandemic and subsequent vaccination suggests that infectious or vaccine-derived immune stimuli may participate in disease initiation in susceptible individuals (18). Intranasal administration of H1N1 virus in mice induces olfactory bulb infection and inflammation, leading to HCRT neuron degeneration and a narcolepsy-like sleep disorder phenotype resembling NT1 (17). Notably, the peak incidence of NT1 occurs in spring and summer, with a sixfold increase compared with winter, further supporting the triggering role of seasonal influenza (19). Animal studies have shown that mice vaccinated with Pandemrix during adolescence exhibit significantly reduced HCRT expression in early adulthood (20). This finding suggests that vaccine exposure may constitute one of the first or subsequent hits to the HCRT system, supporting the hypothesis that the development of NT1 requires cumulative immune or environmental insults. Thus, it is hypothesized that the immune response to Pandemrix vaccination and H1N1 virus infection may jointly induce NT1 through molecular mimicry, whereby structural similarities between microbial antigens and HCRT neuron autoantigens disrupt immune tolerance and trigger autoimmune responses.

Among the molecular pathways through which environmental pathogenic factors breach the central nervous system immune barrier, the olfactory pathway plays a critical mediating role. The core hypothesis proposed by Mori suggests that harmful environmental factors, including pathogens, neurotoxic metal ions, and combustion products, can enter the olfactory bulb via the nasal mucosa, inducing local, persistent neuroinflammation. This leads to abnormally elevated expression of proinflammatory cytokines, such as tumor necrosis factor–alpha (TNF–α) and interferon–gamma (IFN–γ), further disrupting the structural integrity of the blood–brain barrier and facilitating the infiltration of circulating autoreactive T cells into the central nervous system, where they target HCRT neurons (21). These findings indicate that environmental factors likely serve as triggers in narcolepsy.

### 2.3 T-cell-mediated cellular immune attack

T cell-mediated cellular immune responses represent the direct effector mechanism underlying the attack on HCRT neurons. CD4^+^ T cells, as core immune regulatory cells, become activated through antigen recognition and specific interactions with the T cell receptor (TCR). Upon activation, they release large quantities of proinflammatory cytokines, which in turn regulate the proliferation and functional activation of downstream immune effector cells, such as CD8^+^ cytotoxic T cells and B cells, thereby constituting a complete cellular immune attack pathway. Epidemiological evidence suggests an association between influenza A virus infection and the onset of NT1, and a specific fragment of the hemagglutinin (HA) protein derived from the influenza A (H1N1)pdm09 virus has been identified as a potential triggering factor (22, 23). Molecular mimicry studies have shown that HA-derived epitopes share amino acid sequence homology with the carboxyl terminus of the prepro-hypocretin protein (HCRT_NH2_), and cross-reactive T cells targeting these epitopes have been implicated in the autoimmune pathology (24). Recent studies have detected autoreactive T cells that target HCRT neurons in the peripheral blood and cerebrospinal fluid of narcolepsy patients. Furthermore, the selective loss of HCRT neurons in the hypothalamus supports the existence of an autoimmune process targeting these cells specifically (25). Comparative analyses have also revealed increased proportions of CD4+ memory T cells and regulatory T cells (Tregs) in the peripheral blood of NT1 patients compared to the general population, reflecting a more pronounced state of T cell activation (26, 27). However, since CD4+ T cells lack intrinsic cytotoxic capacity and neurons do not express HLA class II molecules, it is unlikely that these cells directly damage neurons (28).Studies have shown that T cells derived from NT1 patients exhibit an enhanced tendency to produce proinflammatory cytokines, particularly with significantly elevated secretion levels of IL-2 and TNF-α, suggesting that inflammatory responses are involved in the pathological process of the disease (29, 30). High sensitivity T cell repertoire analyses have further revealed a significantly increased proportion of autoreactive CD4^+^ T cells in the peripheral blood of narcolepsy patients; these cells can induce local inflammation and compromise blood–brain barrier integrity, thereby enhancing the effector functions of CD8^+^ T cells (31). Under pathological conditions, disruption of the structural integrity of the blood–brain barrier allows autoreactive CD4^+^ T cells to infiltrate the central nervous system, triggering abnormal local immune responses and leading to neuronal damage in the lateral hypothalamic area. Concurrently, infiltrating CD4^+^ T cells and activated microglia promote significant upregulation of various cytokines and chemokines. Moreover, CD8^+^ T cells may target HCRT neurons and further accelerate their death by initiating pro apoptotic signaling pathways (32).Animal models have further confirmed that HCRT neuron-specific CD8^+^ T cells can directly induce neuronal death. Influenza peptides become activated and migrate into the central nervous system. Although CD4^+^ T cells can migrate to the vicinity of neurons, they do not cause damage on their own; however, their synergistic interaction enhances the cytotoxic effect of CD8^+^ T cells (33). Based on the above evidence, the currently proposed molecular mimicry hypothesis posits that, in genetically susceptible individuals exposed to H1N1 influenza antigens, CD4^+^ T cells that recognize DQ0602 influenza peptides become activated and migrate into the central nervous system. Through T cell help, they activate CD8^+^ T cells, which subsequently differentiate into cytotoxic T cells that selectively kill HCRT neurons, ultimately leading to the classic clinical manifestations of NT1.

### 2.4 The role of humoral immune responses

Although most studies tend to support the involvement of humoral immune pathways in narcolepsy type 1 (NT1),evidence for the presence of specific autoantibodies against autoantigens remains lacking.An autoantibody against Tribbles Homolog 2 (TRIB2) was identified in early studies (34). TRIB2 is widely expressed in the central nervous system and even within immune cells. Injection of TRIB2 autoantibody-containing IgG into the lateral ventricles of nude mice induced a narcolepsy-like phenotype, and pathological examination revealed loss of HCRT neurons, neuronal nuclear protein, and synaptophysin in the brain, suggesting that TRIB2 autoantibodies act not only on HCRT neurons (35). However, multiple subsequent studies have not detected any autoantibodies targeting HCRT neurons or IgG sensitive to HCRT precursors or their fragments in patients with NT1. These contradictory findings may reflect differences in detection methodologies, patient selection, or disease stage at sampling. Findings regarding anti-HCRTR2 antibodies are also controversial: anti-HCRTR2 antibodies were significantly elevated in patients with NT1 following Pandemrix vaccination and exhibited cross-reactivity with influenza virus nucleoprotein (36).nevertheless, using a radioligand assay, these antibodies were detected in only 5% of patients, with no significant difference compared with controls (37). These discrepancies likely stem from methodological differences across studies, patient heterogeneity between post-vaccination and sporadic cases, and the possibility that autoantibodies appear transiently and are missed in cross-sectional analyses.Additional autoantibodies, such as those against NEI/αMSH, DP1, NRXN1, GM3, and POMT1, have also been reported, but their positivity rates are low, and they are also present in healthy controls and patients with other sleep disorders, lacking disease specificity (38). The current consensus holds that the humoral immune response in NT1 does not directly mediate HCRT neuron injury, but rather participates in shaping the local inflammatory microenvironment through antigen cross-reactivity induced by molecular mimicry, thereby amplifying the cellular immune effects mediated by CD4^+^/CD8^+^ T cells and synergistically promoting HCRT neuron loss together with cellular immunity (39).Following influenza virus activation, B cells can secrete influenza-specific antibodies that cross-react with HCRT neurons (40). Additionally, patients exhibit elevated titers of non-specific antibodies, such as anti-GM3 and anti-NRXN1, although these antibodies are also detectable in healthy individuals and lack disease specificity (41, 42). During the immune response, the initial immune response triggered by H1N1 influenza antigens can induce local inflammation in hypothalamic tissue and damage HCRT neurons. Self peptides released from damaged tissue are captured and processed by antigen presenting cells and presented to CD4^+^ T cells, subsequently initiating immune responses against other self antigens. Current evidence suggests that the humoral immune response in NT1 does not directly mediate HCRT neuron injury, but rather participates in shaping the local inflammatory microenvironment through antigen cross-reactivity induced by molecular mimicry, thereby amplifying the cellular immune effects mediated by CD4^+^/CD8^+^ T cells and synergistically promoting HCRT neuron loss together with cellular immunity (43). Notably, the humoral immune response induced by the Pandemrix^®^ vaccine differs qualitatively from that induced by natural H1N1 infection, a feature that provides an important immunological basis for explaining the association between vaccination and disease development.

Despite extensive research spanning several years on autoantibodies in narcolepsy type 1, with over 20 candidate antigens identified, no autoantibody has been consistently validated across multiple independent studies to specifically target and destroy HCRT neurons (30).A thorough review of the literature indicates that, although advancements have been made in identifying candidate antigens and developing detection technologies, studies employing gold-standard methodologies have consistently produced negative results. This suggests that many positive findings may be attributed to non-specific binding in solid-phase assays (44).Methodological heterogeneity emerges as the primary factor contributing to data discrepancies. Solid-phase assays, such as ELISA, are susceptible to false positives, whereas the radiobinding assay (RBA) is regarded as the gold standard. The majority of early positive antibody reports originated from ELISA or cell-based platforms; however, subsequent investigations using RBA and protein microarray combined with RBA have failed to replicate these findings (34). Additionally, variations in patient characteristics, including differences in disease duration, vaccination history, and diagnostic confirmation criteria, further contribute to inconsistencies in the research.Nevertheless, there is a lack of longitudinal studies that have monitored antibody dynamics within the same cohort from the acute phase to the chronic phase of the disease, resulting in a complete gap in understanding the evolution of antibody levels throughout the disease course (37, 45).

In summary, current evidence does not support a model in which humoral immunity directly damages HCRT neurons through specific autoantibodies in narcolepsy type 1. Instead, the humoral response likely participates in shaping the inflammatory microenvironment via molecular mimicry, thereby potentiating T−cell−mediated cellular immunity. Three major research gaps need to be addressed: screening for conformation−dependent autoantibodies using gold−standard assays in large NT1 cohorts to determine their true prevalence; longitudinal simultaneous assessment of humoral and cellular immunity to establish their temporal relationship; and direct testing of autoantibody pathogenicity in passive transfer animal models. In contrast, studies on humoral immunity in narcolepsy type 2 are extremely limited, and current evidence has not identified consistent autoantibody abnormalities, suggesting that the immunopathological mechanism of narcolepsy type 2 may be fundamentally different from that of narcolepsy type 1, or may involve other yet−to−be−elucidated immune pathways. The above immune injury ultimately leads to the loss of HCRT neurons, thereby triggering a systemic imbalance in sleep–wake circuits.

## 3 Sleep–wake circuit imbalance and narcolepsy

**Figure 2:**
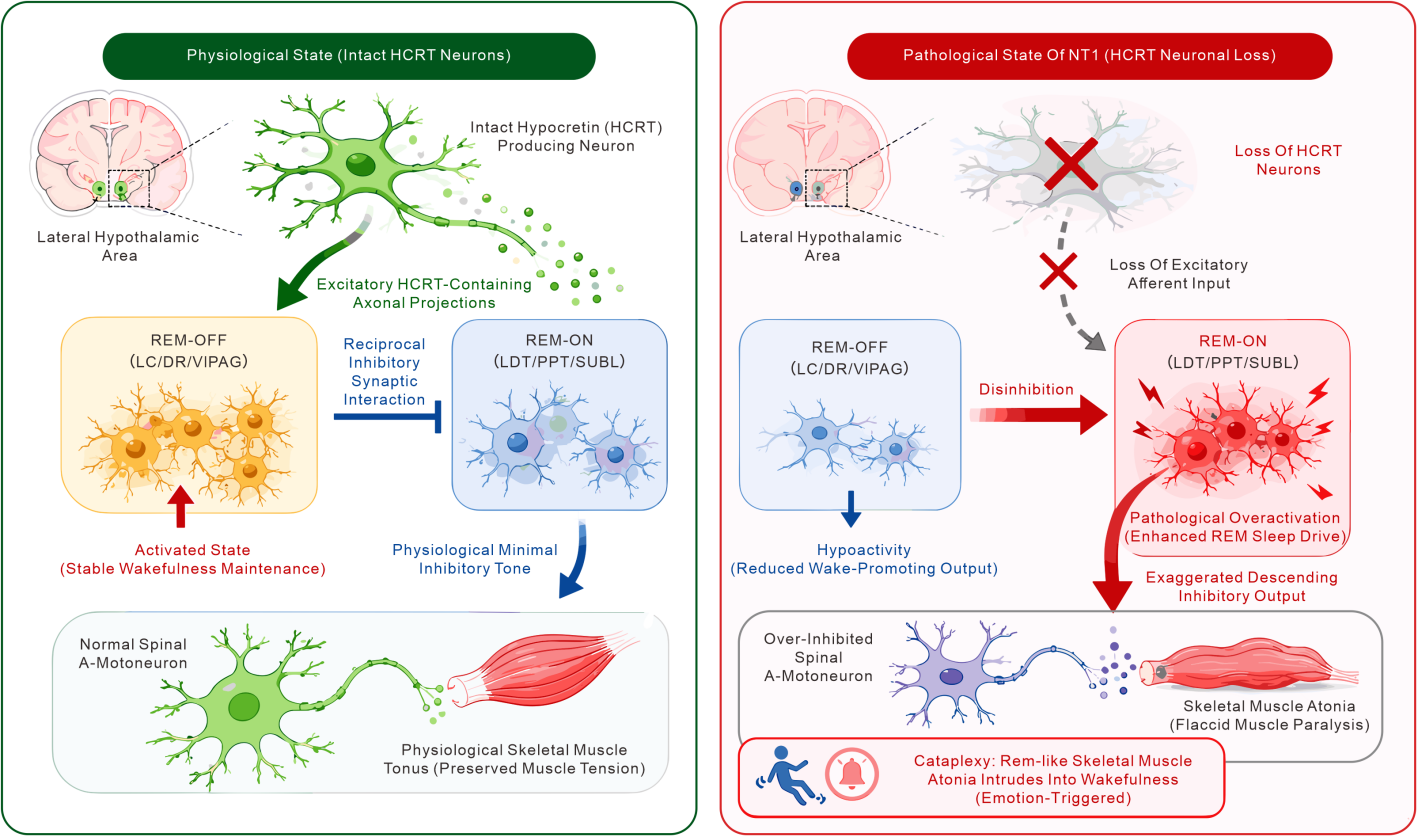
Disrupted sleep-wake homeostatic circuitry accounts for emotion-triggered cataplexy in type 1 narcolepsy (NT1). Left panel (physiological condition): Hypocretin (HCRT) neurons activate REM-off nuclei (locus coeruleus (LC), dorsal raphe (DR), ventrolateral periaqueductal gray (vlPAG)) to inhibit REM-on nuclei (laterodorsal tegmental nucleus (LDT), pedunculopontine tegmental nucleus (PPT), sublaterodorsal nucleus (SubL)). Weak descending signals from REM-on nuclei maintain normal muscle tone of spinal α-motoneurons in wakefulness.Right panel (NT1 pathological state): HCRT deficiency impairs REM-off activity, resulting in overactive REM-on nuclei. Enhanced inhibitory outputs induce wake-time muscle atonia, clinically presenting as emotion-triggered cataplexy.

The specific loss of HCRT neurons is a critical link through which immune injury triggers sleep–wake circuit imbalance. HCRT neurons maintain the stability of wakefulness and the boundary control of REM sleep via widespread projections to arousal promoting nuclei in the brainstem and forebrain. Following the loss of this neuronal population, the sleep–wake circuit undergoes systemic imbalance, manifesting as impaired wake maintenance, loss of REM sleep boundary control, and remodeling of related brain structures and function (**Figure 2**).

### 3.1 Activity and neurotransmitter release patterns of HCRT neurons

The specific loss of HCRT neurons represents the core pathological feature of NT1 (46). Cerebrospinal fluid (CSF) HCRT-1 level is an important biomarker for the diagnosis of NT1, and its diagnostic threshold value—set at below 110 pg/mL—has been clinically validated (47). Of note, reduced HCRT-1 levels are not an absolutely specific marker for NT1, as similar changes can also occur in conditions such as Guillain–Barré syndrome, hypothalamic lesions, and myotonic dystrophy (48, 49).

Although early studies suggested that HCRT is primarily involved in feeding regulation, subsequent research has revealed its critical role in the regulation of sleep–wake rhythms. Furthermore, HCRT serves as a functional mediator linking sleep, appetite, and neuroendocrine function (50).

HCRT neurons typically exhibit higher activity levels during wakefulness (51). HCRT release reaches its peak during wakefulness, particularly during the transition from sleep to wakefulness; in contrast, release levels are at their lowest during sleep and at sleep onset (52). A population of neurons in the hypothalamus projects extensively to cholinergic and monoaminergic nuclei, which in turn project to the cerebral cortex, collectively contributing to the maintenance of wakefulness (53). These neurons broadly innervate monoaminergic nuclei, including the noradrenergic locus coeruleus (LC), the histaminergic tuberomammillary nucleus, the serotonergic raphe nuclei, and the dopaminergic ventral tegmental area (VTA).

### 3.2 Imbalance of the sleep–wake neural circuit

HCRT neurons are closely associated with the sleep–wake circuit. Their fibers project to noradrenergic neurons in the locus coeruleus (LC), serotonergic neurons in the dorsal raphe nucleus (DR), histaminergic neurons in the tuberomammillary nucleus (TMN), cholinergic neurons in the basal forebrain (BF) and pons, and also form fiber connections with the paraventricular thalamus (PVT), the midbrain ventral tegmental area (VTA), and the prefrontal cortex, collectively promoting wakefulness (54, 55).Optogenetic experiments have further confirmed that activating HCRT neurons promotes the transition from sleep to wakefulness, whereas inhibiting their activity significantly increases slow wave sleep, indicating that these cells play a critical role in wake maintenance (56). Loss of HCRT signaling disrupts the stability and activity of the arousal system, resulting in difficulty maintaining wakefulness, which is a key mechanism underlying excessive daytime sleepiness and sleep attacks (57).

Loss of HCRT signaling also reduces norepinephrine and serotonin levels, which are insufficient to activate REM−off neurons. This leads to disinhibition of REM−on neurons and activation of the sublaterodorsal nucleus (SubL), ultimately causing spinal motor neuron dysfunction and decreased skeletal muscle tone, manifesting as cataplexy (58, 59).In the absence of HCRT signaling, MCH neurons are disinhibited and activated, enhancing the propensity for REM sleep, whereas insufficient input from arousal promoting nuclei results in sleep–wake state fragmentation (60).

Sleep-related muscle atonia during wakefulness. The mechanism underlying cataplexy further involves the loss of excitatory regulation by HCRT on the ventrolateral periaqueductal gray (vlPAG), allowing emotional signals from the amygdala and medial prefrontal cortex to abnormally activate the inhibitory pathway from the laterodorsal tegmental nucleus (LDT) to the ventromedial medulla (VMM), thereby inactivating spinal motor neurons (61). Additionally, the disinhibition of noradrenergic neurons in the LC leads to the abnormal initiation of REM sleep-related muscle atonia during wakefulness. Notably, cataplexy episodes are characterized by bursts of theta activity on electroencephalography, suggesting that cataplexy may represent a distinct pathological state, independent of REM sleep (62). Furthermore, the heterogeneous projections of HCRT neurons, disturbances in local hypothalamic microcircuits, and dysregulation of glial cells further exacerbate circuit imbalance, collectively mediating the pathological progression of narcolepsy (63).

### 3.3 Structural brain changes in sleep–wake circuits

Early voxel-based morphometry meta-analyses revealed widespread and consistent gray matter volume reductions in patients with narcolepsy compared with healthy controls, predominantly involving the bilateral hypothalamus, thalamus, nucleus accumbens, anterior cingulate cortex, and frontotemporal cortical regions (64). These structural alterations not only corroborate the core pathological feature of HCRT neuron loss, but also suggest extensive involvement of frontal limbic circuits associated with emotional processing and cognitive function. Diffusion tensor imaging studies have demonstrated reduced fractional anisotropy in the hypothalamus, thalamus, and brainstem regions of patients with narcolepsy, and recent studies have also observed white matter microstructural changes in narcolepsy type 2, suggesting that impaired white matter integrity may have broader pathological significance (65). Functional imaging studies have revealed disrupted functional connectivity in the hypothalamic–thalamic–cortical pathways, particularly among brain regions involved in wake maintenance and REM sleep regulation (66). Proton magnetic resonance spectroscopy (¹H MRS) studies have identified specific alterations in the metabolite profile of the pontine region in patients with NT1, which are closely correlated with disease severity and cerebrospinal fluid HCRT 1 levels (67). These findings suggest that narcolepsy is not merely a focal hypothalamic disorder, but rather a systemic disease involving widespread brain regions.

Hippocampal volume reduction exhibits regional specificity, primarily confined to the anterior portion and the CA1 region (68). Notably, previous studies have not performed subregional comparisons of hippocampal volume between healthy controls and patients with narcolepsy. Specific atrophy of the anterior hippocampus may be associated with cognitive impairment in patients with narcolepsy. Specific atrophy of the anterior hippocampus may be associated with cognitive impairment in patients with narcolepsy. Furthermore, anterior hippocampal volume is negatively correlated with disease duration (69). Although the clinical course of narcolepsy is relatively stable, the progressive reduction in anterior hippocampal volume may reflect persistent sleep architecture disruption and associated neuroplastic changes during disease progression.

## 4 Brain network remodeling and functional instability

**Figure 3:**
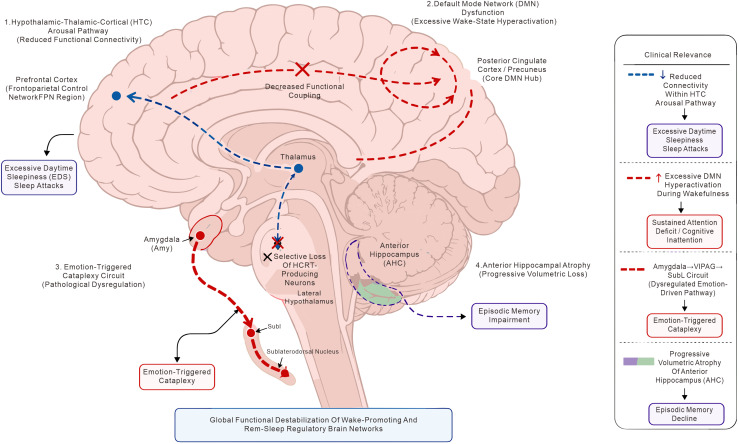
Large-scale whole-brain functional network remodeling underpinning multifaceted clinical phenotypes of type 1 narcolepsy (NT1), displayed on human brain sagittal schematic. Four core pathological network alterations arise from the loss of hypocretin (HCRT) neurons in the lateral hypothalamus: (1) The hypothalamic-thalamic-cortical (HTC) arousal pathway shows weakened connectivity with the frontoparietal control network (FPN), contributing to excessive daytime sleepiness (EDS) and spontaneous sleep attacks. (2) The default mode network (DMN) is overactive during wakefulness and decoupled from FPN, causing attention and cognitive deficits. (3)The amygdala-ventrolateral periaqueductal gray (vlPAG)-sublaterodorsal nucleus (SubL) circuit loses HCRT-related inhibition, leading to emotion-triggered cataplexy. (4) Progressive atrophy of the anterior hippocampus (aHC) is associated with impaired episodic memory.These network disorders disrupt wake and REM sleep regulation, accounting for diverse clinical symptoms of NT1.

The homeostatic regulation of the sleep–wake cycle depends on the coordinated interaction of multiple neural circuits among the brainstem, hypothalamus, and cerebral cortex. When the core regulatory network undergoes neurodegeneration, neurotransmitter imbalance, or abnormal functional connectivity, brain network remodeling is triggered, manifesting as functional instability of the arousal network, loss of boundary control in the REM sleep regulatory network, relay disruption in the hypothalamic–thalamic–cortical pathways, and dysregulation of emotional and cognitive networks, ultimately leading to the typical clinical phenotype of sleep–wake disorders (**Figure 3**).

### 4.1 Functional instability of the arousal network

Rewiring of the arousal network is the primary pathological basis for its functional instability (70). As key nodes of the arousal network, HCRT neurons project densely to all arousal promoting nuclei. Their degeneration leads to a significant loss of excitatory input from the arousal network to the monoaminergic and cholinergic arousal systems. This directly disrupts the antagonistic sleep-wake switching mechanism between the ventrolateral preoptic area and the arousal nuclei. It also substantially impairs the network’s ability to maintain cortical arousal levels and vigilance (71). Furthermore, it triggers functional abnormalities between the arousal network and cortical functional networks. These abnormalities manifest as excessive activation of the default mode network (DMN) during wakefulness and significantly reduced functional connectivity between the frontoparietal control network (FPN) and the DMN (72, 73).This remodeling—from brainstem–hypothalamic regulatory circuits to higher-order cortical functional networks—ultimately results in the characteristic features of arousal network functional instability. These features are manifested as excessive daytime sleepiness, sleep attacks, and sustained attention deficits (74).

### 4.2 Loss of boundary control in the REM sleep regulatory network

The REM sleep regulatory network consists of the REM-off region, comprising the LC, DR, and vlPAG, and the REM-on region, comprising the LDT, pedunculopontine tegmental nucleus (PPT), and SubL. These two regions form a flip-flop switch through mutual inhibition, jointly regulating the initiation and termination of REM sleep (75).In the absence of HCRT signaling, excitatory input to The REM-off region is reduced, which leads to the disinhibition of the REM-on region. Decreased levels of norepinephrine and serotonin are insufficient to activate REM-off neurons, allowing REM-sleep-related muscle atonia to abnormally intrude into wakefulness (76–78). Degeneration of GABAergic and glycinergic inhibitory pathways in regions such as the ventromedial medulla (VMM) leads to the failure of motor inhibition signaling mediated by the subcoeruleus nucleus (SubC). This results in impaired network regulation of skeletal muscle atonia, which manifests as abnormal dream-related motor behaviors during REM sleep (79). Reduced monoaminergic inhibition of cholinergic circuits, combined with loss of arousal network regulation following HCRT neuron loss, results in the failure of the ventrolateral periaqueductal gray (vlPAG) to exert negative constraint on the subcoeruleus nucleus (SubC). The boundary between the REM sleep regulatory network and the arousal network becomes disrupted. Abnormal activation signals within the network penetrate wakefulness and manifest as clinical symptoms, such as cataplexy. Cataplexy represents the abnormal intrusion of REM sleep features into wakefulness (80–83). This loss of the boundary between the REM sleep regulatory network and the arousal network, induced by network remodeling, represents a core pathological feature of conditions such as REM sleep behavior disorder and narcolepsy (84).Furthermore, abnormal activation of spinal motor neuron inhibition via brainstem inhibitory pathways by emotional signals from the amygdala and medial prefrontal cortex explains why cataplexy is frequently triggered by emotional stimuli (85, 86). Loss of boundary control in the REM sleep regulatory network manifests as not only cataplexy, but also sleep paralysis and hypnagogic hallucinations. These symptoms collectively reflect the abnormal persistence of REM sleep components at the wake–sleep boundary (87).

### 4.3 Relay disruption in the hypothalamic–thalamic–cortical pathway

The hypothalamic–thalamic–cortical (HTC) pathway is responsible for transmitting sleep–wake signals to the cerebral cortex, and functional instability of this pathway plays a critical role in brain network remodeling in narcolepsy (88). In patients with NT1, there is severe and selective loss of HCRT neurons in the hypothalamus. These neurons project extensively to thalamic nuclei and upstream regulatory regions, providing stable excitatory drive to the thalamus through the release of HCRT and the co-transmitter glutamate (89, 90). HCRT deficiency leads to a marked reduction in excitatory input to the thalamus and disrupts the modulatory effects of HCRT on the histaminergic and monoaminergic systems, further compromising upstream drive (91, 92). As a key relay hub of the HTC pathway, the thalamus, through its non-specific thalamocortical projection system, is responsible for broadly transmitting sleep–wake signals to the cortex (93). In patients with NT1, the absence of HCRT-mediated excitatory regulation results in decreased thalamic synaptic transmission efficiency and impaired synchrony of neuronal activity (94).Neuroimaging studies have confirmed significantly reduced functional connectivity between the thalamus and regions such as the prefrontal cortex in patients (95), along with structural reductions in thalamic gray matter volume, suggesting that both anatomical and functional connections between the thalamus and cortex undergo remodeling (96, 97). These alterations disrupt the coupling between REM sleep slow-wave activity and REM-related muscle atonia, further exacerbating sleep fragmentation and brain network instability (98, 99).

### 4.4 Dysregulation of emotional and cognitive networks

Reconstruction of the emotional network centers on abnormalities in the amygdala–hypothalamus–brainstem pathway. Loss of excitatory regulation by HCRT neurons on the central amygdala, basolateral amygdala, and bed nucleus of the stria terminalis leads to destabilized boundary control between the emotional center and the REM sleep regulatory network. Positive emotional or reward related stimuli can excessively inhibit the vlPAG via GABAergic neurons in the central amygdala, thereby relieving inhibition of the sublaterodorsal nucleus (SubL) in the pons and triggering skeletal muscle atonia during wakefulness, manifesting as cataplexy (100, 101). In patients with NT1, reduced amygdala volume, metabolic abnormalities, and diminished functional connectivity with the postcentral gyrus and occipital lobe further contribute to impaired emotional regulation and increased risk of comorbid conditions, such as anxiety and depression (102).

Reconstruction of the cognitive network is primarily characterized by functional deficits in the prefrontal cortex and associated circuits. HCRT deficiency results in loss of excitatory drive from the monoaminergic and cholinergic systems to the prefrontal cortex, accompanied by reduced gamma oscillations and an elevated GABA/glutamate ratio, leading to decreased attention, executive dysfunction, and working memory impairment (103). White matter structural damage between the DMN and the frontoparietal control network (FPN) further exacerbates cognitive dysfunction (104). Abnormal functional connectivity between the anterior cingulate cortex and the frontal and occipital lobes correlates with anxiety and depression scores (105). Cognitive deficits and emotional dysregulation—both stemming from loss of HCRT regulation—together with functional instability of the arousal network and relay disruption in the hypothalamic–thalamic–cortical pathway constitute the core features of global functional instability in the brain network in narcolepsy (106, 107).

## 5 Conclusion and prospects

The pathogenesis of narcolepsy involves an autoimmune response mediated by cellular and humoral immunity that selectively destroys HCRT neurons in genetically susceptible individuals following exposure to environmental triggers. This leads to neural circuit imbalance and functional instability of brain networks. However, the precise identification of the autoantigens and the specific mechanisms underlying T-cell-mediated pathology remain to be elucidated, and the mechanisms of narcolepsy type 2 are still poorly understood.

The prevailing linear model — immune trigger → HCRT neuron loss → circuit dysfunction → network remodeling — provides a useful heuristic framework but may oversimplify the complex pathogenesis of narcolepsy. While not yet experimentally validated, one might speculate that pre-existing functional vulnerabilities within specific brain networks could theoretically lower the threshold for immune-mediated targeting of HCRT neurons. This possibility warrants future investigation. Additionally, chronic sleep-wake fragmentation resulting from HCRT loss could theoretically induce sustained low-grade neuroinflammation, which might in turn create a feed-forward loop that perpetuates immune cell activation or blood-brain barrier dysfunction. Addressing these questions will require longitudinal studies combining neuroimaging, immune profiling, and clinical phenotyping.

Several concrete research directions emerge from these considerations. First, the identification of primary autoantigens is crucial. Unbiased screening technologies such as HLA-peptide multimer assays and single-cell TCR sequencing could pinpoint autoantigenic targets in recent-onset patients. Second, if a dominant autoantigen is found, targeted immunotherapies including tolerizing vaccines and engineered regulatory T cells may provide effective treatments with fewer side effects than general immunosuppression. Third, there is an urgent need for peripheral biomarkers to identify at-risk individuals before significant neuron loss occurs and to track treatment responses. Potential biomarkers include disease-specific T-cell clonotypes and serum neurofilament light chain, the latter indicating neuronal injury. Fourth, long-term strategies such as HCRT neuron replacement using induced pluripotent stem cells and viral vector-mediated HCRT gene delivery are promising but face challenges in targeted delivery and safety. Fifth, narcolepsy type 2, which lacks HCRT deficiency, needs research into other neurotransmitter systems such as melanin-concentrating hormone and GABAergic circuits. Addressing these questions will require extensive multi-center studies with deep phenotyping, immune monitoring, and neuroimaging.

In summary, future research should focus on identifying key target antigens, clarifying the cellular and humoral immune mechanisms by which immune cells attack HCRT neurons, investigating narcolepsy-related susceptibility genes and environmental factors, and further elucidating the relationship between environmental factors, such as influenza virus infection and vaccination, and disease development. This will provide a theoretical basis for developing effective interventions.
